# Quantifying the free energy landscape between polymers and minerals

**DOI:** 10.1038/s41598-017-09041-3

**Published:** 2017-08-17

**Authors:** K. K. Sand, R. W. Friddle, J. J. DeYoreo

**Affiliations:** 1 Physical Sciences Division, Pacific Northwest National Laboratory, Richland, WA USA; 2 Sandia National Laboratories, Livermore, California 94550 USA; 3 Department of Materials Science and Engineering, University of Washington, Seattle, WA 98195 USA; 4 Nano-Science Center, Department of Chemistry, University of Copenhagen, Denmark and Geography & Earth Sciences, Aberystwyth University, Aberystwyth, United Kingdom

## Abstract

Higher organisms as well as medical and technological materials exploit mineral-polymer interactions, however, mechanistic understanding of these interactions is poorly constrained. Dynamic force spectroscopy can probe the free energy landscape of interacting bonds, but interpretations are challenged by the complex mechanical behavior of polymers. Here we restate the difficulties inherent to applying DFS to polymer-linked adhesion and present an approach to gain quantitative insight into polymer-mineral binding.

## Introduction

Exploiting favorable interactions between biopolymers and mineral surfaces is a vital strategy used by organisms to enhance the strength of skeletal structures and control mineral growth processes. Bioinspired approaches to materials design and synthesis utilized such interactions to advance medical and technological applications, such as nanoparticles for ingestion, bone implants, and responsive materials. However, these processes and technologies rely upon molecular-scale mechanisms about which little is known. Dynamic force spectroscopy (DFS) can be used to quantify both the free energy (∆G_bu_) and kinetic parameters associated with bonds between molecules and materials, thus providing fundamental insights into underlying mechanisms. However, polymer-linked DFS present unique challenges when extracting quantitative information from the raw data. There are three main difficulties: 1) Biopolymers typically exhibit internal dissipative processes resulting in a non-linear dependence of extension on applied force which presents analytical challenges to modeling the experiment using the linear DFS model^1,6,7,9,10^. 2) It is a challenge to decipher the amount of interacting bonds when an unknown number of bonds are clustered at the probe-surface contact^1^. 3) The equilibrium regime for polymer-based DFS is often overlooked, and an accepted approach to extracting ∆G_bu_ from slow polymer-linked rupture data is non-existent. Consequently, various approaches have been taken to obtain bond properties of complex polymers^2–5^ without a clear consensus on the appropriate method.

In DFS, bond rupture forces (*f*_*r*_) are determined by retracting a sharp, functionalized AFM tip from a surface and measuring the cantilever deflection at the point of rupture. When the rate of loading (*r*) is systematically varied at discrete, constant rates, an analytic function for the mean rupture force vs loading rate can be used for data fitting^6^. Both theory and experiment have demonstrated^7–9^ that when loading rates are slow enough to occur on a timescale similar to that of bond breaking and re-forming, a near-equilibrium regime is probed and an equilibrium rupture force (*f*_*eq*_) can be extracted. As the loading rate is increased, the rupture behavior transitions into an irreversible kinetic regime where the intrinsic unbinding rate *k*_*off*_ and distance to the transition state *x*_*t*_ can be recovered from the data^7^.

## Results

### Obtaining meaningful parameters from the DFS data

**Figure 1:**
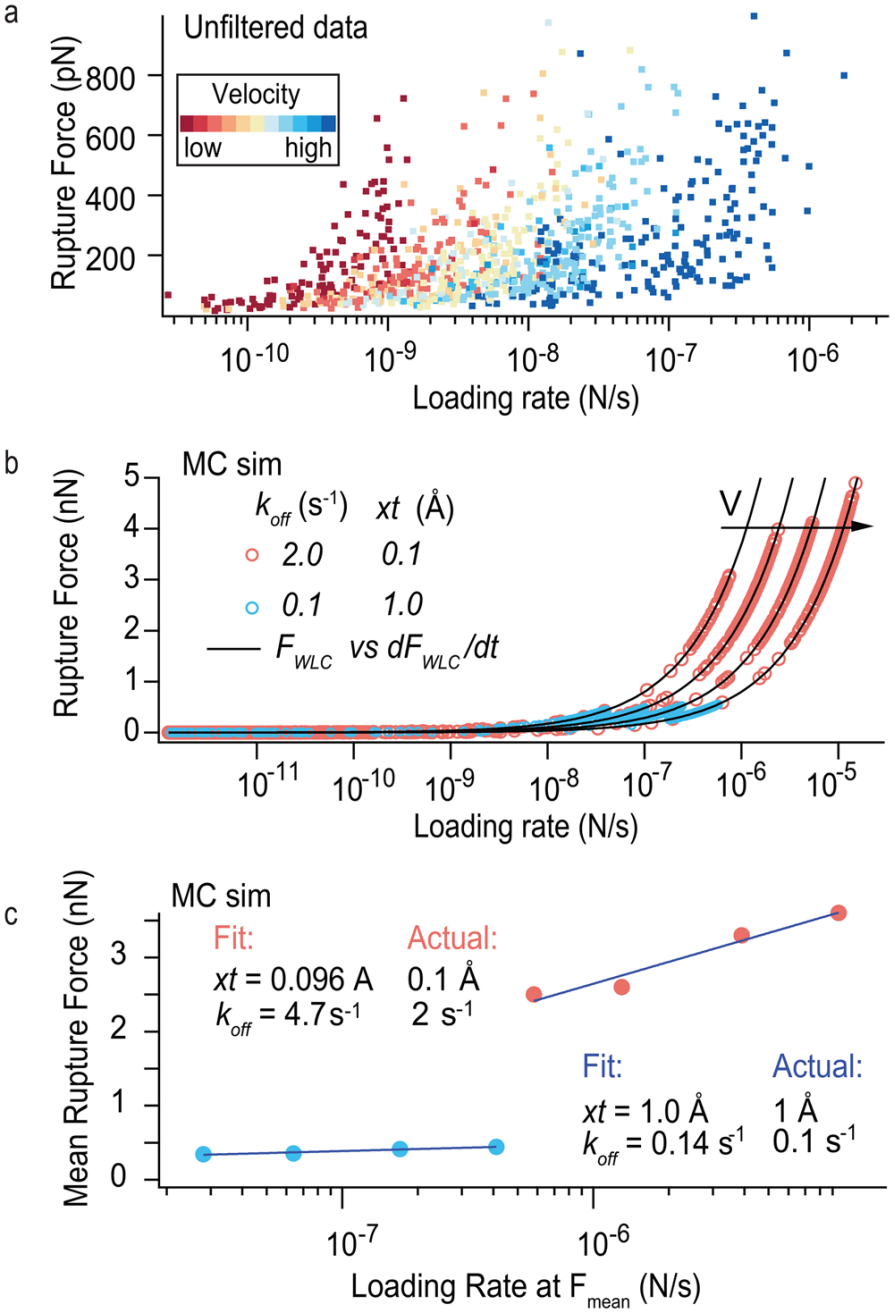
Examples of analyzing polymer-based DFS data. (**a**) All rupture-forces vs loading-rate at rupture pairs for the retraction velocities. (**b**) Monte Carlo simulation of rupturing two different bonds (blue dots and red circles) using the same polymer linker (i.e. same WLC parameters). The arrow indicates data sets of increasing retraction velocity. (**c**) Data from (**b**) plotted as mean rupture force vs mean loading rate, with fits of the linear DFS model. Fit parameters and a discussion of errors are given in Supplementary Table S1.

In the standard DFS protocol^9, 10^, the bond is interrogated at a single loading rate, *r*, for a given retraction velocity (*v*). This is the case when the bond is linked directly to a probe that exhibits constant stiffness over the range of forces applied, as is the case for an AFM cantilever. When a polymer links the tip to the bond, the polymer stiffness changes with the applied force, and hence the bond is swept over a band of loading rates starting from the soft entropic regime of the polymer coil and ending in the stiff elastic regime of the fully extended chain (Fig. 1a). For a given retraction velocity (color codes in Fig. 1a), stochastic rupture of the bond occurs at forces distributed across this band of loading rates and it is a challenge to fit the linear DFS model to the non-linear polymer extension. This spread of rupture events over a continuum of loading rates can deceptively appear as a complete dataset of rupture force versus loading rate from a single retraction velocity. However, when the data is presented this way, the resulting force spectrum is simply the representation of the polymer’s force versus its own time derivative – it has no physical relevance to the underlying bond. We illustrate this in Fig. 1b,c where Monte Carlo simulation of two differently parameterized bonds pulled via the same polymer extension parameters (Equation 3–4 **)** results in data that fall precisely on the same force versus time derivative of force for the worm like chain (WLC) function. In contrast, when the data are plotted as mean rupture force vs mean loading rate the errors involved in using the linear model to approximate the data is minimized and a fit of the linear DFS model^1, 6, 7, 9, 10^ approximates the actual parameterized bonds significantly better than by fitting the spread of individual force vs loading rate events (Fig. 1c, Supplementary Table S1) as also shown by Friedsam *et al*.^8^.

To obtain *f*_*r*_ for one molecular interaction, from an uncontrolled number of interacting polymers, the last rupture event of a force-extension trajectory is fitted to a model of the polymer chain’s extension with force (e.g. WLC^11, 12^ or freely joined chain^13, 14^). This fitting provides a measure of the last rupture event’s stiffness through the measured (apparent) persistence length (*l*_*app*_), which can be related to the number of molecules (*N*) on the tip through *N* = *l*_*p*_/*l*_*app*_, where *l*_*p*_ is the single polymer persistence length.

### Single bond method

**Figure 2:**
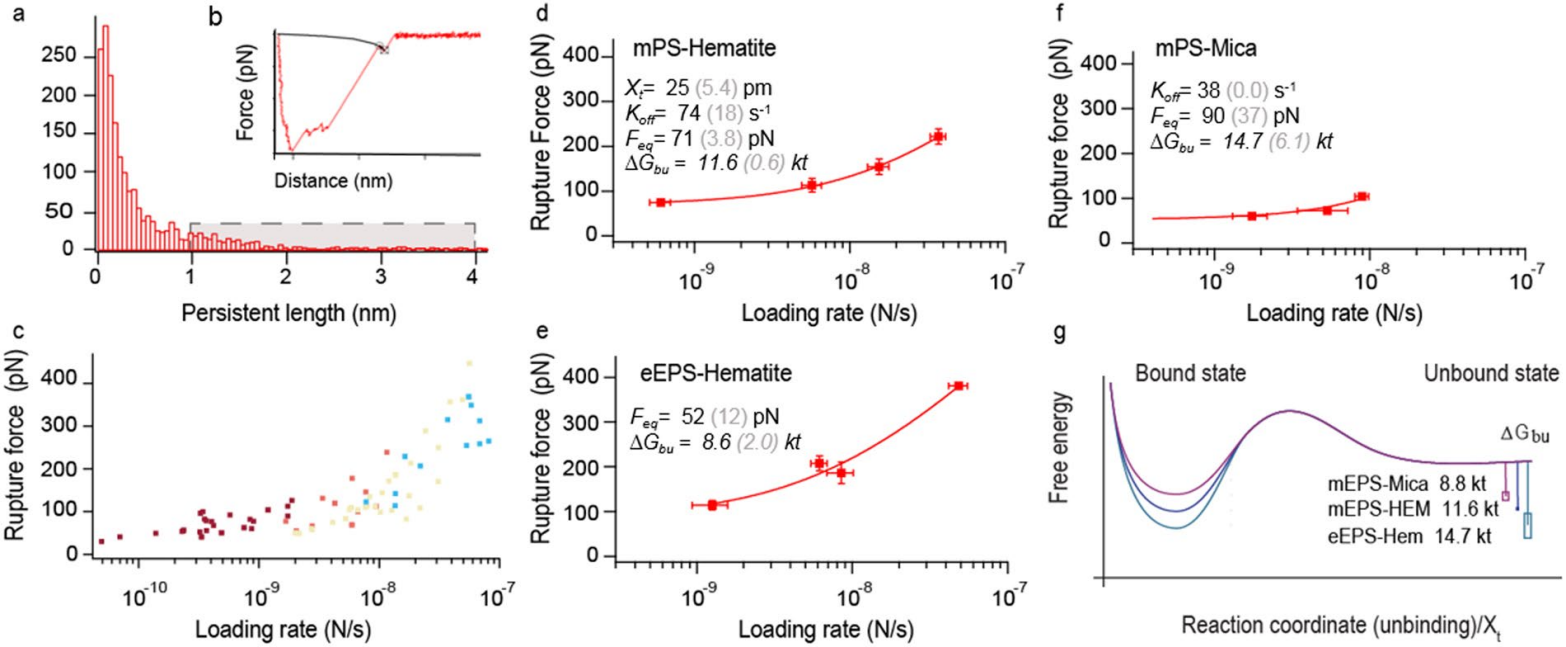
Analyzing polymer-based DFS data. (**a**) Histogram of *l*_*p*_ showing the block of filtered values in grey. (**b**) WLC fit to a force curve. (**c**) Data in Fig. 1a filtered for persistence lengths. Color coding show the velocity groups used for obtaining the mean *r* and *l*_*r*_. (Fig. 2d). (**d**–**f**) *f*_*r*_ vs *r* for the EPS-mineral interactions. (**g**) Free energy landscape.

To surmount these challenges to interpreting DFS data for polymers, we demonstrate an appropriate protocol (Fig. 2) by investigating mineral binding by biopolymers in microbial systems. Microbes bind to minerals through charged groups on their extracellular polymeric substances (EPS). Natural EPS mainly consists of polysaccharides but also contain small amounts of lipids, DNA and proteins^15^. We covalently fixed EPS extracted from *S. oneidensis* (eEPS) and a model EPS (mEPS) (alginate) to an AFM tip. Both types of EPS have an overall negative charge where the functional groups of mEPS only consist of negatively charged carboxyl groups, where the eEPS has both negatively charged phosphate groups and positively charged amine groups (Supplementary Fig. S1 and Table S2). We collected force curves from contact with the commonly occurring minerals hematite and mica (model for clay). We defined *r* as *dF*/*dt* (N/s) taken directly from the force-time trajectory of the polymer and *f*_*r*_ as the measured rupture force. We obtain single molecule-mineral interaction by fitting the last rupture event (Fig. 2a) to the WLC model and used only those events with *l*_*app*_ above a threshold set near the single-polymer persistence length (Fig. 2b). The filtering reduces the amount of relevant data points (compare Figs 1a and 2c). Data representative of the statistics of rupture were then obtained in same manner as the MC data in Fig. 1c, by averaging the *l*_*app*_-filtered data for *r* and *f*_*r*_ taken at similar velocities (red dots in Fig. 2c–e). The experimental values for *f*_*eq*_, *x*_*t*_, and *k*_*off*_ (Fig. 2. Supplementary Table S3) were extracted by fitting the final data to the single bond model^6^ as delineated in the Methods section.

### Free energy of binding

The equilibrium regime where the forces tends to plateau, *f*_*eq*_ is rarely considered in polymer-based DFS. Manohar *et al*.^16^ showed that, at equilibrium, the rupture force required to pull a polymer away from a surface is related to the adhesion free energy per unit length *γ*_*adh*_ by,

1 $${\gamma }_{adh}=\frac{{k}_{B}T}{b}\,\mathrm{ln}(\frac{4\pi \,\sinh (\frac{{f}_{eq}b}{{k}_{B}T})}{\frac{{f}_{eq}b}{{k}_{B}T}})$$

where *b* is the Kuhn length of the polymer (*b* = 2*l*_*p*_). When the product of the equilibrium force with the Kuhn length is much larger than the thermal energy, *f*_*eq*_ *b*/*k*_*B*_ *T*  ≫ 1, equation (1)) reduces to, *γ*_*adh*_ ≈ *f*_*eq*_. It then follows that the free energy of binding per monomer (∆*G*_*bu*_) is related to the energy of the polymer chain as,

2 $${\rm{\Delta }}{G}_{bu}={\gamma }_{adh}{l}_{mono}\approx {f}_{eq}{l}_{mono}$$

where *l*_*mono*_ is the expected length of our monomer (0.675 nm), which is the sum of the length of a saccharide ring (0. 483 nm)^17^ and the length of the carboxyl group expected to be involved in the bonding, (0.192 nm). The significance of the quantity *f*_*eq*_ *l*_*mono*_ is that it balances the free energy of a monomer between the surface-bound and solution-suspended configurations.

## Conclusion

Our results show that ${\rm{\Delta }}{G}_{bu}$ is more favorable between EPS and hematite than between EPS and mica. This is not surprising considering EPS is dominated by negative functional groups (PO_4_, and COO-) and mica and hematite are negatively and positively charged, repectively. The value of ${\rm{\Delta }}{G}_{bu}$ between EPS and hematite is larger for eEPS than for mEPS, implying a higher equilibrium coverage for eEPS, but interestingly the kinetic barrier to bond breaking is greater for mEPS implying a slower rate of desorption and faster exchange kinetics for mEPS. In contrast to the mEPS, we cannot know which combination of the possible functional groups are responsible for the probed bond behavior for the eEPS. However, the data gives an overall picture of the thermodynamic landscape representing the strongest interactions for both polymers.

This formulation of a DFS_polymer_ approach applies to both the equilibrium and nonequilibrium regimes; it should be adapted to describe the bond behavior and binding mechanisms of elastic (bio)polymers and can add to the applicability of the DFS method to complex biopolymers.

## Materials

*The chemicals* used for tip functionalization were reagent grade compounds, purchased at Sigma Aldrich and used as received. Solutions were prepared using ultrapure deionized water (Milli-Q, resistivity > 18.2 MΩ·cm^−1^). The mEPS is a Pronova UP LVM alginate purchased from Novamatrix, Norway. The eEPS was the loosely bound fraction extracted from *Shewanella oneidensis* strain MR-1 biofilms^18^.

We used the *tip functionalization protocol* from Sletmoen *et al*. where EPS were covalently attached to sharpened Bruker silicon nitride MSCT tips with a nominal spring constant k = 0.01–0.6. Briefly, we rinsed the AFM tips thoroughly using 3 sequences of ethanol and MilliQ water. After drying, they were plasma cleaned for 10 minutes, followed by silanization in freshly prepared 1% (v/v) solution of trimethoxysilylpropyl-diethylenetriamine and 1 mM acetic acid, for 20 min at room temperature. Subsequently they were rinsed in MilliQ. We used 1-(3-dimethylaminopropyl)-3-ethylcarbodiimide hydrochloride (EDAC) as a coupling agent^4, 19, 20^ between the amino groups of the silanized AFM tips and the carboxylate on the polysaccharide. We incubated 0.5 mg/mL EDAC for 1 h with 20 *μ*g/mL EPS in 50 mM boric acid, pH 5.8. The solutions for the DFS measurements were prepared from 10 mM NaCl and MilliQ. All solutions were filtered through a 0.22 μm filter prior to use.

*The minerals used* were the {001} face from a polished, single crystal of hematite (HEM) as a substrate for DFS measurements. The face was cleaned in 1 M NaOH, rinsed in MilliQ water and plasma cleaned for 10 min prior to use. The mica (grade V-3) was purchased at Ted Pella Inc. and freshly cleaved prior to use.

## Methods

*Dynamic Force Spectroscopy (DFS)* measurements were made at 20 **°**C using an Asylum MFP3D atomic force microscope. We used the minimum trigger force possible (in general, 120 pN) to avoid damage to the tip during the force measurements. We determined the true cantilever spring constant at the end of the measurements using the thermal calibration method, at a trigger point of 2,500 pN. Subsequently, the tip was discarded. The DFS measurement was setup to use a constant approach rate of 500 nm/s, a dwell time of 1 second and collection of minimum 100 force curves for seven retraction speeds for each experiment (5 nm/s to 10 µm/s). The effect of local mineral heterogeneities was minimized by changing the retraction speed every 5 force curves, while the tip was probing random points on the surface. A minimum of 20 such cycles was made for each experiment, resulting in at least 700 force curves per experiment.

*The worm like chain model (WLC)* was used to discriminate between multiple and single rupture events. We fitted the model to the last rupture event of each force curve to estimate the number of interacting molecules (*N*) involved in the rupture.

3 $$\frac{f{l}_{app}}{{k}_{B}T}=\frac{x}{L}+\frac{1}{4{(1-\frac{x}{L})}^{2}}-\frac{1}{4}$$

4 $${l}_{app}=\frac{{l}_{p}}{N}$$

where *f* represents the force, *l*_*app*_ the apparent persistence length, *L* the contour length of the polysaccharide, *x*, the tip-surface separation and *l*_*p*_, the persistence length of a single polymer. The *l*_*app*_ represents the steepness of each force curve. It decreases with increasing number of polymers participating in the adhesion cluster. Thus, measurements with the highest recurring persistence lengths most closely correspond to a single polymer and therefore most likely represent a single molecule rupture event.

### Single bond model

*f*_*eq*_, the equilibrium force, *x*_*t*_, distance between transition states, and *k*_*off*_, intrinsic unbinding rate, were determined by fitting the filtered and averaged data for *r* and *f*_*r*_ to the single bond model^6^:

5 $$\langle f\rangle \cong {f}_{eq}+{f}_{\beta }{e}^{\frac{1}{R({f}_{eq})}}{E}_{1}(\frac{1}{R({f}_{eq})}),$$

where

6 $$R({f}_{eq})=\frac{r}{{k}_{u}({f}_{eq}){f}_{\beta }\,},$$

7 $${E}_{1}(z)={\int }_{z}^{\infty }\frac{{e}^{-s}}{s}ds,$$

8 $${k}_{u}(f)={k}_{off}{e}^{f/{f}_{\beta }}$$

and where *k*_*u*_ *(f)* is the unbinding rate *and f*_*β* =_ *k*_*B*_ *T/x*_*t*_, and *r* represents the loading rate.

*Monte Carlo (MC) Simulations* were carried out over four retraction velocities (*v* = 0.1, 0.21, 0.46, 1.0 μm/s) for each of two single-bond types discussed in the text. At each time step Δ*t* (1 μs), the force of a WLC polymer model (equation 3) was calculated at an extension *x* = *vt*. The probability of rupture at this force and time is calculated as

9 $${P}_{u}={k}_{u}(f){\rm{\Delta }}t,$$

where *k*_*u*_(*f*) is the Bell unbinding rate (equation 8). A random number generator (Igor Pro) uniformly distributed over [0,1] is used to produce a random number *ξ*. When *P*_*u*_ > *ξ*, a rupture takes place. For each pulling velocity 300 rupture events were recorded.

## Electronic supplementary material

Supporting information

## References

[1] Sulchek T, Friddle RW, Noy A (2006). Strength of Multiple Parallel Biological Bonds. Biophys. J..

[2] Alsteens D (2015). Imaging G protein-coupled receptors while quantifying their ligand-binding free-energy landscape. Nat. Methods.

[3] Tao J (2015). Energetic basis for the molecular-scale organization of bone. Proc. Natl. Acad. Sci..

[4] Sletmoen M, Skjåk-Braek G, Stokke BT (2004). Single-molecular pair unbinding studies of Mannuronan C-5 epimerase AlgE4 and its polymer substrate. Biomacromolecules.

[5] Bowman KA, Aarstad OA, Stokke BT, Skjåk-Bræk G, Round AN (2016). Sliding Contact Dynamic Force Spectroscopy Method for Interrogating Slowly Forming Polymer Cross-Links. Langmuir.

[6] Friddle RW, Noy A, De Yoreo JJ (2012). Interpreting the widespread nonlinear force spectra of intermolecular bonds. Proc. Natl. Acad. Sci..

[7] Friddle RW, Podsiadlo P, Artyukhin AB, Noy A (2008). Near-Equilibrium Chemical Force Microscopy. J. Phys. Chem. C.

[8] Friedsam C, Wehle AK, Kühner F, Gaub HE (2003). Dynamic single-molecule force spectroscopy: bond rupture analysis with variable spacer length. J. Phys. Condens. Matter.

[9] Evans E, Ritchie K (1997). Dynamic strength of molecular adhesion bonds. Biophys. J..

[10] Evans E (1999). Introductory Lecture Energy landscapes of biomolecular adhesion and receptor anchoring at interfaces explored with dynamic force spectroscopy. Faraday Discuss..

[11] Kratky O, Porod G (1949). Röntgenuntersuchung gelöster Fadenmoleküle. Recl. Trav. Chim. Pays-Bas.

[12] Bustamante C, Marko JF, Siggia ED, Smith S (1994). Entropic elasticity of lambda-phage DNA. Science.

[13] Flory PJ, Volkenstein M (1969). Statistical mechanics of chain molecules. Biopolymers.

[14] Lee N-K, Thirumalai D (2004). Pulling-Speed-Dependent Force-Extension Profiles for Semiflexible Chains. Biophys. J..

[15] Cao B (2011). Contribution of extracellular polymeric substances from Shewanella sp. HRCR-1 biofilms to U(VI) immobilization. Environ. Sci. Technol..

[16] Manohar S, Jagota A (2010). Sequence-dependent force response during peeling of single-stranded DNA from graphite. Phys. Rev. E Stat. Nonlin. Soft Matter Phys..

[17] Williams MAK, Marshall A, Haverkamp RG, Draget KI (2008). Stretching single polysaccharide molecules using AFM: A potential method for the investigation of the intermolecular uronate distribution of alginate?. Food Hydrocoll..

[18] Cao B (2011). Extracellular polymeric substances from Shewanella sp. HRCR-1 biofilms: characterization by infrared spectroscopy and proteomics. Environ. Microbiol..

[19] Gelinas S, Finch JA, Vreugdenhil AJ (2000). Coupling of diethylenetriamine to carboxyl-terminated magnetic particles. Colloids Surf. -Physicochem. Eng. Asp..

[20] Yang B, Yang BL, Goetinck PF (1995). Biotinylated hyaluronic acid as a probe for identifying hyaluronic acid-binding proteins. Anal. Biochem..

