# Supplementary material for: Quantifying the free energy landscape between polymers and minerals
Source: Sci Rep. 2017 Aug 17;7:8663. doi: 10.1038/s41598-017-09041-3 (PMC5561072; doi:10.1038/s41598-017-09041-3)
Supplement: Supplementary file 1 — Supporting information [file 41598_2017_9041_MOESM1_ESM.pdf]

Quantifying the free energy landscape between polymers and minerals

K.K. Sand[1†], R.W. Friddle[2] and J.J. DeYoreo[1,3]

Affiliations:
[1] Physical Sciences Division, Pacific Northwest National Laboratory, Richland, WA, USA.
[2] Sandia National Laboratories, Livermore, California 94550, USA.
[3] Department of Materials Science and Engineering, University of Washington, Seattle, WA, USA 98195

*Correspondence to: kks@nano.ku.dk, rwfridd@sandia.gov

† Current addresses: Nano-Science Center, Department of Chemistry, University of Copenhagen, Denmark and Geography & Earth Sciences, Aberystwyth University, United Kingdom.

Supporting information

Supplementary Table 1. Fit parameters using different approaches a) MC simulations and b) mEPS-HEM data

a

**MC sim**	x_t (pm)	k_{off} (s^{-1})		x_t (pm)	k_{off} (s^{-1})
Example of bonds	1	1		2	2
Actual parameters	10	2		100	0.1
Fit individual	5.5 ± 0.3	38.7 ± 6.4		71.2 ± 1.7	1.4 ± 0.2
Fit mean	9.6 ± 1.5	4.8 ± 4.4		103.8 ± 17.03	0.1 ± 0.2

b

**mEPS-Hem**	x_t (pm)	k_{off} (s^{-1})	f_{eq} (pN)
Fit filtered-mean	25 ± 5.4	74 ± 18	71 ± 3.8
Fit filtered-individual	16 ± 5.3	72 ± 22	57 ± 8.2
Fit unfiltered-individual	53.8 ± 6.7	6.6 ± 3.1	101 ± 1.5

Individual: The single bond model are fitted to all the individual measurements. Mean: the single bond method are fit to mean values for r and l_r based on velocity groupings. Unfiltered: All obtained data points. Filtered: data points above a l_p threshold, approximating a single bond interaction.

EPS vs PS composition. Alginate is a linear PS consisting of linked pyranose rings, where each ring has one carboxyl group (COO^-). It is a linear polysaccharide with covalently linked blocks of (1→4)-linked b-D-mannuronate (M) and C-5 epimerα-L-guluronate (G) residues. Alginate is a quite common PS in natural systems and is often used as a model for bacterial EPS. Composition differs with bacteria species but in general, EPS consists of polysaccharides, proteins and lipids. Two forms can be isolated from *S. oneidensis*: bound (bEPS) and loosely associated (laEPS), where the latter is thought to facilitate metal binding and the former is considered an extension of the cell membrane so has a higher ratio of proteins and redox active compounds [15]. The ratio of polysaccharide to proteins in laEPS is 3.6 and for bEPS it is 0.8 [15]. Fourier transform infrared spectroscopy (FTIR) patterns of the alginate and laEPS used in this work [18] confirm that the alginate is a pure sugar and the EPS consists of sugar, lipids and proteins.

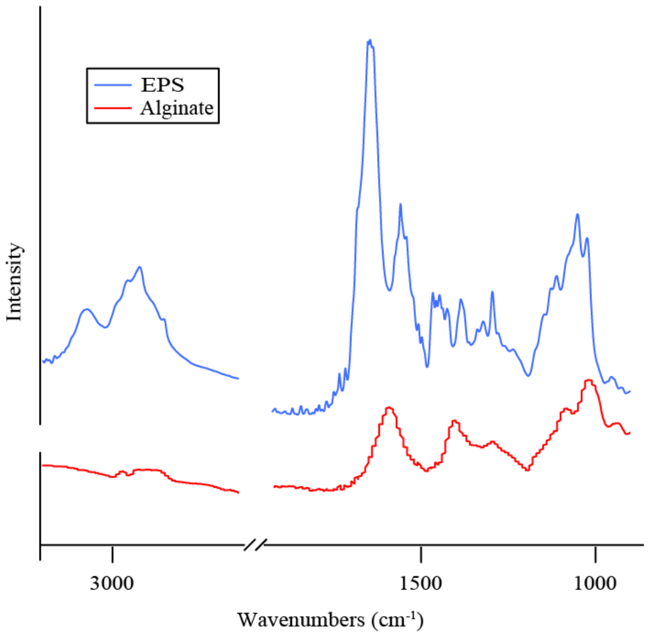

Supplementary Figure 1. FTIR spectra for alginate and purified *Shewanella* EPS.

Supplementary Table 2. FTIR peaks observed in Fig.1.

**Vibrations (cm^{-1})**	**Vibrations (cm^{-1})**	**Assignment**
Alginate	EPS	
3000-3700	3000-3700	O-H stretch
	2850	-CH- vibrations in lipids
2890		CH stretch
	1653	Amide 2 protein
1590		Asymmetric stretch COO^- stretch
	1560	Amide 1 protein
1400		Symmetric
	1383	O-H deformation and C-O stretch
	1326	Skeletal vibration
	1300	Skeletal vibration
1290	1290	Skeletal vibration
1020-1090	1020-1090	Symmetric C-O-C stretch

Supplementary Table 3. Fit parameters using the DFS$_{polymer}$ protocol

**DFS data**	x_t (pm)	k_{off} (s^{-1})	f_{eq} (pN)	ΔG_{bu} (kt)
mEPS-Mica		38 ± 0	90 ± 37	14.7 ± 6.1
rEPS-Hem			52 ± 12	8.6 ± 2.0
mEPS-Hem	25 ± 5.4	74 ± 18	71 ± 3.8	11.6 ± 0.6
